# A Microfluidic Platform for Viscosity Testing of Non-Newtonian Fluids in Engineering and Biomedical Applications

**DOI:** 10.3390/mi17020201

**Published:** 2026-02-01

**Authors:** Yii-Nuoh Chang, Da-Jeng Yao

**Affiliations:** 1 Institute of NanoEngineering and MicroSystems, National Tsing Hua University, Hsinchu 300, Taiwan; 2 Department of Power Mechanical Engineering, National Tsing Hua University, Hsinchu 300, Taiwan

**Keywords:** non-Newtonian fluids, microfluidic chip, viscosity measurement, dairy gelation

## Abstract

This study presents a microfluidic platform for non-Newtonian fluid viscosity sensing, integrating a high-flow-rate flow field stabilizer to mitigate flow uniformity limitations under elevated flow rate conditions. Building upon an established dual-phase laminar flow principle that determines relative viscosity via channel occupancy, this research aimed to extend the measurable viscosity range from 1–10 cP to 1–50 cP, which covers viscosity regimes relevant to biomedical fluids, dairy products during gelation, and low-to-moderate viscosity industrial liquids. A flow stabilizer was developed through computational fluid dynamics simulations, optimizing three key design parameters: blocker position, porosity, and the number of outlet paths. The N5 design proved most effective, providing over 50% reduction in standard deviation for asymmetric velocity distribution in high-flow simulations. The system was validated using simulated blood and dairy samples, achieving over 95% viscosity accuracy with less than 5% sample volume error compared to conventional viscometers. The chip successfully captured viscosity transitions during milk acidification and gelation, demonstrating excellent agreement with standard measurements. This low-volume, high-precision platform offers promising potential for applications in food engineering, biomedical diagnostics, and industrial fluid monitoring, enhancing microfluidic rheometry capabilities.

## 1. Introduction

In recent years, non-Newtonian fluids have found increasingly widespread applications across diverse fields such as medical diagnostics, the food industry, and biopharmaceutical development [1,2,3,4]. Characterized by a viscosity that varies with shear rate, these fluids exhibit complex rheological behaviors that play a critical role in both process performance and functional outcomes [5,6,7]. Understanding their shear-dependent viscosity is essential for precise system control and design optimization.

In the dairy sector, for instance, monitoring the viscosity of milk during acidification and casein coagulation provides valuable insight into gelation behavior, which is critical for product consistency and quality [8,9]. Conventional rheometers offer precise shear viscosity data, but their high cost, operational complexity, maintenance requirements, and large sample volume demand limit their practical use in fast-paced or resource-limited research and industrial environments [10,11].

While traditional viscometers are more affordable and user-friendly, they are typically limited to single-point measurements and cannot capture the full shear-dependent behavior of non-Newtonian fluids [12,13]. This makes them inadequate for comprehensive rheological characterization. In biomedical applications, non-Newtonian viscosity monitoring is particularly relevant for blood viscosity screening, point-of-care diagnostics, and low-shear characterization of biofluids, where sample volume is limited and conventional rheometers are impractical.

Over the past decade, microfluidic technology has rapidly advanced, offering advantages such as low sample volume requirements, flexible operation, and easy integration with sensors and control systems [14,15,16]. These features provide a new pathway for fluid property measurements. Compared with conventional viscometers, microfluidic platforms enable precise control of shear-rate distributions through channel geometry, facilitate numerical prediction via CFD, and allow the integration of auxiliary structures for flow-field regulation. These features make microfluidics particularly suitable for non-Newtonian viscosity measurements under low-shear and multi-shear-rate conditions. In this context, the development of a microfluidic chip capable of accurately analyzing non-Newtonian viscosity under multiple shear rates, while minimizing sample consumption, has become a significant research focus in modern rheological sensing. Previous iterations of our non-Newtonian viscosity sensing platform demonstrated good accuracy and reproducibility within the viscosity range relevant to blood measurement [17]. However, the limited measurement range restricted its broader applicability beyond biomedical samples. Previous work by our group established a two-phase laminar flow platform capable of accurate viscosity measurement in the 1–10 cP range. At higher flow rate ratios, however, that design exhibited velocity-field instability, which limited the system’s measurable range and reproducibility. While most microfluidic viscometers focus on blood-relevant viscosities (1–10 cP), several important applications require characterization of fluids up to approximately 50 cP [10,17,18,19,20]. This includes clinically defined swallowing-safe liquids, early-stage dairy gelation processes, and a wide range of industrial fluids such as cosmetic toners, lotions, inkjet inks, and pharmaceutical formulations. In these contexts, viscosity monitoring below 50 cP remains critical for process control before strong viscoelastic or yield-stress behavior dominates. To overcome this limitation, the present study integrates a high-flow-rate flow-field stabilizer to enhance flow uniformity and measurement stability, thereby extending the detectable viscosity range up to 50 cP [17]. The relative viscosity relationship is defined as follows [21]:

$$\mu_{s}=\frac{Q_{r}}{Q_{s}}\cdot \mu_{r}\cdot \frac{N_{s}}{N_{r}}$$

$\mu_{s}$ and $\mu_{r}$ denote the viscosities of the sample and reference fluids, respectively; $Q_{s}$ and $Q_{r}$ represent their corresponding flow rates; and $N_{s}$ and $N_{r}$ are the number of indicator microchannels fully occupied by each fluid.

This phenomenon is mainly attributed to interfacial shear force [22,23]. When one fluid flows faster than the other, a velocity gradient near the interface generates viscous shear stress [24,25]. Such local velocity differences also translate to local kinetic energy discrepancies, which—according to Bernoulli’s principle—lead to pressure redistribution. This pressure gradient further drives or impedes the flow locally, ultimately distorting the symmetry and stability of the overall velocity field [26].

Several strategies of flow distributor have been proposed to mitigate such flow instability in microfluidic systems:

- Bifurcated networks based on Murray’s law to achieve uniform fluid division [27,28,29];
- Geometric regulation, such as tapered inlets or stepped distribution channels [30,31,32];
- Auxiliary structures, such as inserting baffles at the inlet to adjust flow distribution [33,34,35].

While bifurcation and geometric approaches perform well under low flow rate conditions, their effectiveness diminishes as inertia becomes significant at higher flow rates. Moreover, increasing the number of bifurcations leads to higher system pressure loss, which is impractical for industrial-scale applications requiring large-scale parallelization. In contrast, auxiliary elements, positioned externally at the chip inlet, do not alter the original chip design and are better suited for high-flow scenarios, making them an attractive solution for flow field stabilization.

However, when extending microfluidic viscometers to higher viscosity ranges, increased flow rate ratios often induce flow-field distortion and velocity asymmetry, resulting in large measurement variability and poor repeatability. Addressing this stability issue is therefore critical for expanding the practical applicability of microfluidic viscosity sensing platforms. The primary objective of this work is to systematically address this flow-field-induced instability and its impact on measurement repeatability. In this study, we address the velocity field distortion resulting from expanded measurement ranges, by integrating an optimized flow distributor at the chip inlet. We apply this improved platform to dairy-related viscosity monitoring, utilizing a microfluidic design capable of controlling shear rate distribution. The proposed system aims to deliver accurate and stable measurements across low to moderate viscosity fluids, thereby broadening the scope of applicable fluids and operating conditions for the microfluidic rheometry platform.

Moreover, achieving stability and reproducibility in microfluidic viscosity measurements is equally critical. The standard deviation (SD) of repeated trials provides a direct quantitative indicator of flow-field stability and repeatability. Conventional viscometers seldom report SD due to large sample volumes and limited low-shear capability, whereas microfluidic systems are more sensitive to temperature drift and pump precision. Therefore, improving SD performance becomes a primary design target in this study. The following sections demonstrate that the integration of a flow stabilizer not only expands the measurable viscosity range (1–50 cP) but also reduces SD by more than 50%, providing a stable platform for both biomedical and food-engineering samples.

## 2. Materials and Methods

### 2.1. Principle of Viscosity Measurement

The viscosity measurement in this study is based on a previously established microfluidic method involving two-phase parallel laminar flow, as detailed in our prior work. In this approach, a reference fluid of known viscosity and a sample fluid of unknown viscosity are simultaneously introduced into a microchannel structure comprising a transient region and a microarray sensing zone.

Under fully developed laminar flow, both fluids experience the same pressure drop across the microarray zone. By leveraging the hydraulic analogy and assuming dominant resistance in the sensing zone, the relative viscosity of the sample can be determined from the observed flow partitioning. In the simplified form, the relationship can be expressed as:

(1) $$\mu_{s}=\frac{Q_{r}}{Q_{s}}\cdot \mu_{r}\cdot \frac{N_{s}}{N_{r}}$$

where $\mu_{s}$ and $\mu_{r}$ are the viscosities of the sample and reference fluids, $Q_{s}$ and $Q_{r}$ are the respective flow rates, and $N_{s}$ and $N_{r}$ are the number of occupied microchannels for each fluid in the array region.

In the present work, this principle is retained but further extended to high-flow-rate conditions through the integration of a flow distributor. The flow distributor design improves interface stability and velocity field symmetry, which are critical under high flow rate ratios for maintaining accurate channel indexing and minimizing measurement error.

### 2.2. Computational Setup

Numerical simulations were conducted using COMSOL Multiphysics 6.3, employing the Laminar Flow (spf) module to solve the incompressible Navier–Stokes equations. Although some non-Newtonian fluids may exhibit transient or time-dependent rheological behavior, the present study focuses on steady laminar flows under constant flow-rate conditions, where the velocity field rapidly reaches a fully developed state. Therefore, steady-state Navier–Stokes equations were adopted to capture the dominant flow characteristics relevant to viscosity measurement. A stationary (steady-state) study was adopted to analyze flow behavior under various flow rate ratios. Second-order discretization was used for spatial derivatives to ensure solution accuracy. The computational domain included the inlet region, flow distributor, sensing channels, and outlet structures, and was meshed using a combination of free tetrahedral and swept hexahedral elements, resulting in a mesh count of approximately 1.2 million elements. Mesh independence was verified by conducting refinement studies, ensuring that the computed flow profiles and outlet velocities remained consistent across different mesh densities [17]. In addition to flow-rate variations, representative viscosity values within the investigated range were also examined to confirm that the mesh resolution was sufficient to capture velocity gradients without affecting simulation accuracy.

In the CFD simulations, a power-law model was employed to represent non-Newtonian fluid behavior. The apparent viscosity was defined as a function of local shear rate, with model parameters selected to match the experimentally measured viscosity range relevant to blood analog fluids and early-stage dairy samples [17]. This approach allows the simulation to capture shear-rate-dependent flow characteristics without introducing additional time-dependent rheological effects.

To ensure numerical stability and consistency across all simulations, the governing physical models, material properties, and boundary condition settings were kept identical throughout the parametric studies. These simulation parameters are summarized in Table 1.

### 2.3. Design Strategy of Flow Distributor

**Figure 1:**
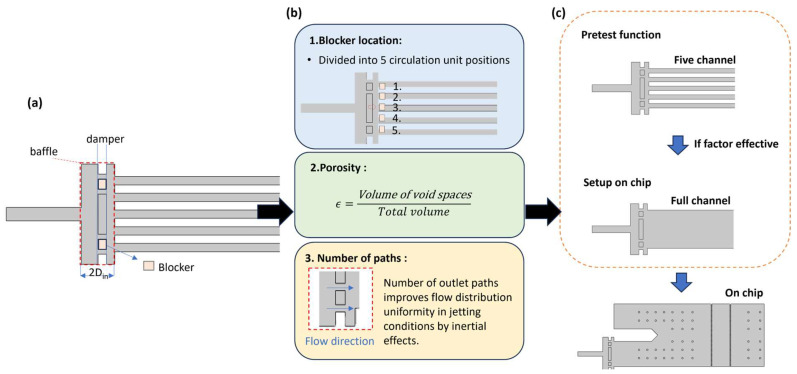
Structural design and simulation workflow of the flow distributor. (**a**) Schematic illustration of the flow distributor structure. (**b**) Key design factors and geometric parameters of the flow distributor. (**c**) Simulation workflow, in which segmented simulations were conducted to analyze the evolution of the flow field.

In this study, an auxiliary structure approach was adopted for flow distributor design. Specifically, we referenced the baffle-based flow distribution methodology described in Young-June et al. [34]. Three key design parameters were identified and optimized, as summarized in Figure 1:

- Blocker position—controls the location and magnitude of the peak outlet velocity, thereby regulating flow partitioning across channels. The baffle is too far from the inlet (at 3/4 of the damper chamber height): The fluid has over-diffused and cannot be effectively distributed; the baffle is too close to the inlet (at 1/4 of the damper chamber height): The fluid rebounds after hitting the baffle, resulting in non-uniform flow [34].
- Porosity—defined as the ratio of open area between the inlet and outlet, ranging from 0 to 1; optimal flow uniformity was observed when porosity was maintained between 0.4 and 0.6 [36].
- Number of flow paths—determines the degree of lateral symmetry and overall velocity uniformity at the outlet.

To clearly define and verify these design guidelines within the context of our microfluidic chip, a series of CFD simulations were performed based on the original chip layout. The simulation parameters were established using previously validated experimental conditions for simulated blood, and the flow rate ratio was systematically increased from 1:1 to 1:10 in increments of 1.

These parameters collectively establish the geometric framework for optimizing flow-field balance under high-flow-rate conditions. The structural design and simulation workflow are summarized in Figure 1, where segmented CFD analyses were conducted to evaluate the influence of each parameter on flow-field evolution.

### 2.4. Microfluidic Device Preparation Protocols

**Figure 2:**
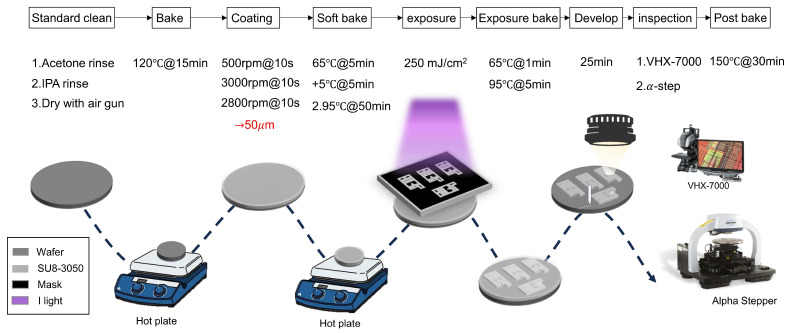
Process flow diagram of the photolithography procedure. The fabrication steps proceed from left to right, with individual process details listed from top to bottom within each step.

The microfluidic chip is replica with a typical soft lithography technique. A polydimethylsiloxane (PDMS; Sylgard 184 A/B, Dow Corning Corporation, Midland, MI, USA) was irrigated to the silicon mold with typical mixing ratio of Sylgard 184 A and B [17]. The silicon mold was fabricated by patterning channel array structures in the sensing region using a negative photoresist, SU-8 3050 (SU-8 3050, Kayaku Advanced Materials, Westborough, MA, USA) on a silicon wafer, as shown in Figure 2.

**Figure 3:**
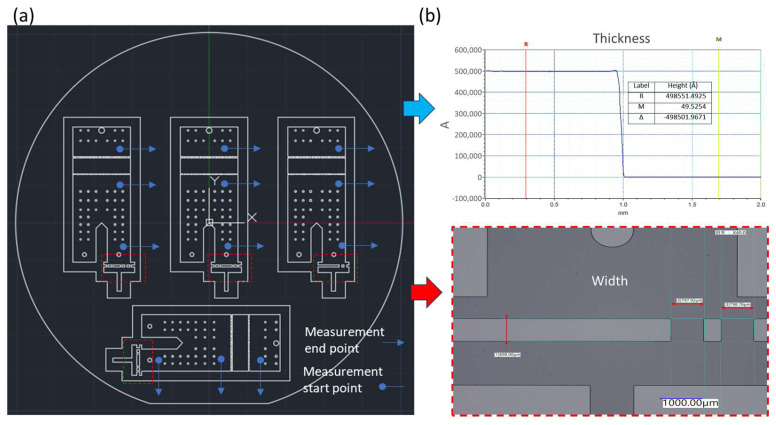
Structural characterization of SU-8 microfeatures. (**a**) Measurement layout and reference positions. (**b**) Results of thickness and linewidth measurements.

The internal channel height was designed to be 50 μm. Channel thickness was verified using a film profilometer (α-step, Bruker, MA, USA), yielding an average thickness of 49.89 ± 1.68 μm under spin-coating conditions of 3000 rpm. To ensure fabrication accuracy, dimensional measurements were conducted at designated inspection points within the array area using a digital optical microscope (VHX-7000, Keyence, Japan). The deviation between designed and fabricated dimensions was found to be less than 5%, which was deemed acceptable for this study as shown in Figure 3.

### 2.5. Sample Preparation Protocols

For blood analog testing, a simulated blood solution was prepared following the manufacturer’s specified formulation, resulting in a fluid viscosity of 4.85 ± 0.05 cP. This is consistent with the fluid properties used [17].

Milk samples used in this study were sourced from commonly available domestic commercial brands to minimize batch-to-batch viscosity variation. Whole milk and low-fat milk from Fresh Delight and Lin Feng Ying were stored at 4 °C and tested within 24 h of opening. For coagulation experiments, the transformation of milk from liquid to semi-solid phase was induced by acidification using 3 wt% glucono delta-lactone (GDL, Dyi Chemical, Taiwan). The acidification process followed the methodology reported by Kruif et al. [20], and viscosity changes during coagulation were monitored simultaneously using two approaches: (1) bulk measurement in a 20 mL beaker using a vibrating-type viscometer, and (2) The viscosity monitoring within the microfluidic chip using a continuous 0.5 mL sample injection. The results from both methods were compared for validation.

## 3. Results and Discussion

### 3.1. Flow Field Distortion Under High Flow Rate Ratios

It should be noted that the present study focuses on flow-field stability and interface behavior as the primary observables for viscosity extraction. The viscosity values are obtained through an integrated relationship between velocity field and shear-rate-dependent viscosity, rather than direct spatial mapping. As such, velocity distributions are presented to illustrate flow uniformity and symmetry, which directly govern measurement accuracy and repeatability. The local viscosity field is therefore implicitly accounted for through the governing equations and post-processing formulation.

**Figure 4:**
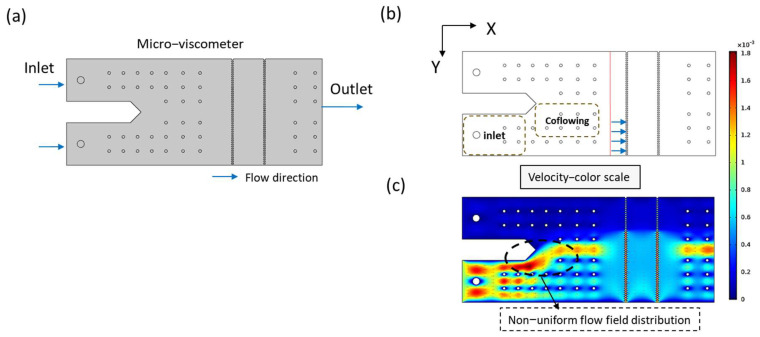
CFD configuration and simulation results illustrating flow field distortion as the flow rate ratio increases (unit: m/s). (**a**) Schematic diagram of the simulated chip structure. (**b**) Velocity distribution near the inlet and co-flowing region; the red line indicates the position where the velocity profile is extracted. (**c**) COMSOL−simulated velocity contour showing localized flow concentration.

As discussed in the introduction, extending the measurable viscosity range requires increasing the flow rate ratio. However, simply increasing the flow rate ratio disrupts the internal flow field stability of the microfluidic chip. Computational fluid dynamics (CFD) simulations show that as the flow rate ratio increases from 1:1 to 1:10, significant velocity distribution asymmetry emerges, as shown in Figure 4.

**Figure 5:**
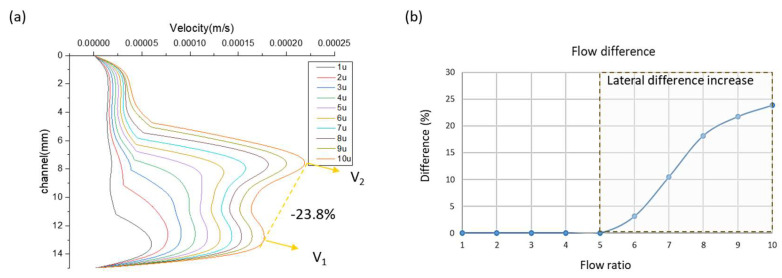
Cross-sectional velocity profiles corresponding to flow rate ratios ranging from 1:1 to 1:10. (**a**) Velocity profiles extracted along the red cross-sectional line indicated in Figure 1b, illustrating the CFD-simulated flow velocity distribution at each flow rate ratio. (**b**) Relationship between flow rate ratio and lateral velocity difference, showing that flow non-uniformity increases proportionally once the ratio exceeds 1:5.

As shown in Figure 5, when the flow rate ratio exceeds 1:5, the velocity profile becomes non-uniform, forming a characteristic bimodal distribution. To quantify the degree of lateral velocity non-uniformity, we employed an interpolation-based flow lateral difference analysis, defined as:

(2) $$Flow\;lateral\;difference=100\%\times \frac{V_{2}-V_{1}}{V_{1}}$$

where $V_{2}$ is the peak velocity at the co-flow center and $V_{1}$ is the maximum velocity near the sidewall. This metric reflects the extent of flow asymmetry caused by the bimodal structure. A direct correlation was observed between increased flow rate ratio and flow non-uniformity, with the maximum deviation reaching 23.8%. This instability affects the interface oscillation and the accuracy of interface positioning, thereby compromising the measurement repeatability.

### 3.2. The Geometry of Design Simulating Result

To reduce simulation time and computational cost, each design concept was first subjected to preliminary simulations individually. These pre-simulations aimed to assess the detailed flow rate distribution of each model. Once the optimal configuration for flow improvement was identified, a comprehensive simulation incorporating the full chip model was conducted to evaluate overall performance, as illustrated in Figure 1.

The evaluation of performance focused on the degree of flow field deviation in the lower half of the cross-section. This is based on the design intent of utilizing controlled flow field offset to compensate for the inherent non-uniformity in the velocity distribution.

#### 3.2.1. Relative Position of the Blocker at the Inlet and Outlet

**Figure 6:**
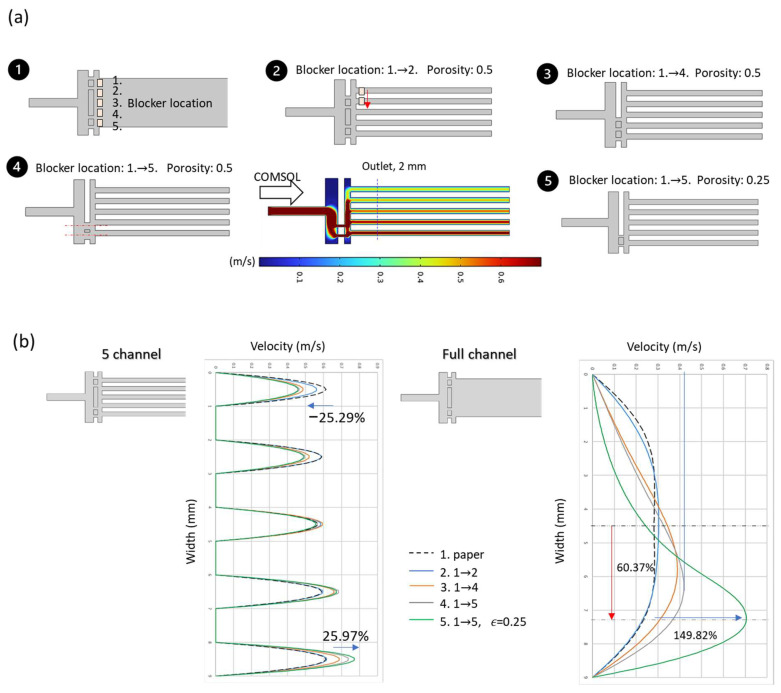
Flow field simulations of blocker positioning. (**a**) Designs 1–5 with different blocker configurations. (**b**) Simulated velocity profiles at the outlet region. The five-channel flow distributions clearly reveal trends in flow partitioning under each design.

We established five different flow distributor models and fine-tuned both the blocker position and porosity, as shown in Figure 6a. Adjusting the relative position of the blocker proved effective in altering the flow distribution across the microchannel cross-section, with simulation results clearly illustrating this effect as shown in Figure 6b.

As the blocker position was shifted downward, the originally symmetric flow distribution gradually became biased toward the lower channels. A comparison between the symmetric configuration (Design 1) and the most offset configuration (Design 5) reveals a ~25% decrease in flow rate through the upper channels, mirrored by a ~25% increase in the lower channels. Furthermore, Design 5 achieved a 5% greater enhancement compared to Design 4 the second-best configuration confirming the effectiveness of blocker positioning in modulating flow distribution.

We then performed localized chip simulations incorporating the selected flow distributor to observe the shift in maximum velocity location within the cross-section. From Designs 1 to 4, which varied only in blocker positioning, the maximum velocity shifted laterally by up to 45% across the cross-section, with an increase in peak velocity of nearly 50%. These results demonstrate that adjusting the relative position of the blocker effectively compensates for flow field asymmetry.

Design 5 introduced a combined strategy—employing the maximum blocker offset while reducing porosity by 50%. This configuration led to a 60% shift in the velocity peak location and a 150% increase in peak velocity, highlighting the synergistic impact of both blocker position and porosity as design factors.

Based on these promising results, we proceeded to carry out full-chip flow field simulations for Designs 4 and 5.

#### 3.2.2. Adjustment of Porosity

**Figure 7:**
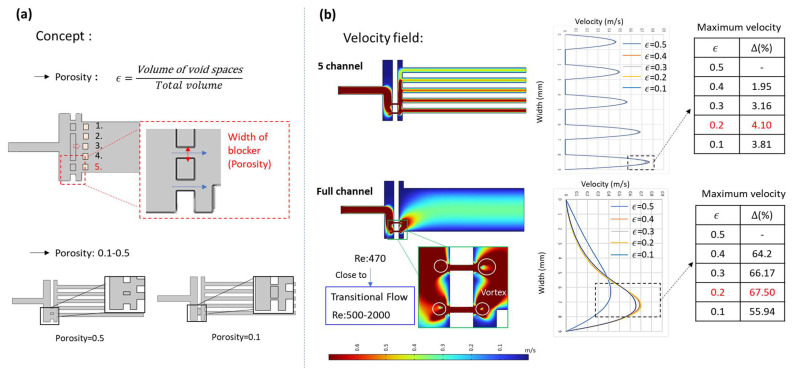
Flow field simulation results for porosity variation. (**a**) Structural designs with porosity values from 0.1 to 0.5. (**b**) Simulated outlet velocity profiles across five microchannels.

Building upon the previous section, where Design 4 was identified as producing the most significant flow field deviation through blocker positioning, we proceeded to evaluate the impact of porosity adjustment based on the same blocker configuration. As shown in Figure 7a, the structural design was modified to achieve porosity values ranging from 0.1 to 0.5 by altering the blocker width, while keeping the relative blocker position constant.

As illustrated in Figure 7b, five-channel simulations under different porosity configurations revealed that the maximum velocity was achieved with a porosity of 0.2. This design yielded a 4.1% increase in peak velocity compared to the highest-porosity case (0.5). When compared to the previously evaluated porosity of 0.25, the 0.2 case exhibited only a marginally lower velocity enhancement, suggesting that peak velocity does not increase monotonically with decreasing porosity. Beyond a porosity value of 0.25, further reduction led to a consistent decline in peak velocity. Full-chip CFD simulations revealed that when the porosity was reduced to 0.2, vortex formation was observed near the narrowed throat region of the flow distributor. This was attributed to the excessively constricted channel width, which caused the local Reynolds number to approach the transitional flow regime.

#### 3.2.3. Number of Path Adjustment

**Figure 8:**
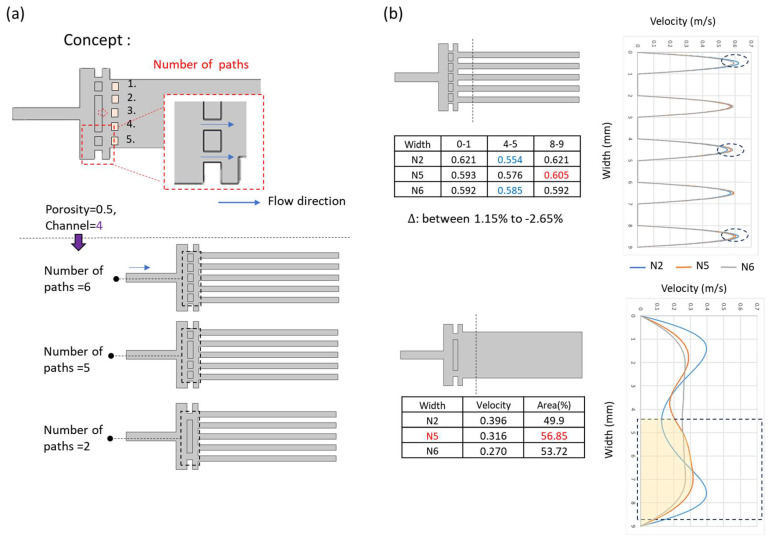
Simulation results for flow path number adjustment. (**a**) Structural designs with three different numbers of flow paths. (**b**) Simulated velocity profiles at the outlet region, with the dashed line indicating the measured cross-sectional plane.

To investigate the influence of the number of flow paths on velocity distribution, we extended the previously symmetric structural concept and proposed three designs with different flow path counts, as shown in Figure 8a.

As illustrated in Figure 8b, the predictive simulations revealed that among the three designs, the configuration labeled N2 produced the highest peak velocity, with a 1.15% increase compared to the initial design. However, its velocity distribution exhibited a clear bimodal structure, indicating minimal effectiveness in compensating for flow field asymmetry.

In contrast, the N6 design demonstrated the most uniform flow distribution across all outlet paths, suggesting strong symmetry but limited correction of interface deviation. The N5 configuration exhibited a distinct lateral shift in the velocity profile across different cross-sectional zones, with localized velocity enhancements of 3–6% compared to the other two configurations. This indicates that N5 offers superior compensation for flow field deviation among the three tested designs.

Based on these findings, the N5 design was selected for full-chip CFD simulation to further evaluate its performance under realistic operating conditions.

### 3.3. Full-Chip Flow Field Simulation Results

**Figure 9:**
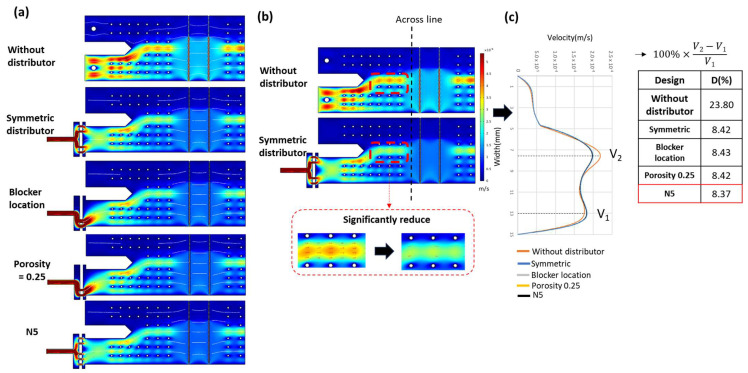
Evaluation of flow field compensation effectiveness by flow distributors. (**a**) Full-chip flow field simulation results for different flow distributor configurations. (**b**) Velocity field improvements based on CFD simulation trends. (**c**) Quantified compensation efficiency among different designs.

Based on the results of previous pre-simulations and localized chip analyses exploring various design concepts for flow field manipulation, we selected four distinct flow distributor configurations for full-chip CFD simulation, as shown in Figure 9. 

As shown in Figure 9b, the compensation effect of the flow distributor is clearly observed. While localized velocity concentration still exists near the chip inlet region, it is significantly mitigated compared to the baseline case without a flow distributor. In the coflowing region, the velocity near the fluid interface is also substantially reduced, indicating effective suppression of interfacial shear dominance and improved flow field balance.

**Figure 10:**
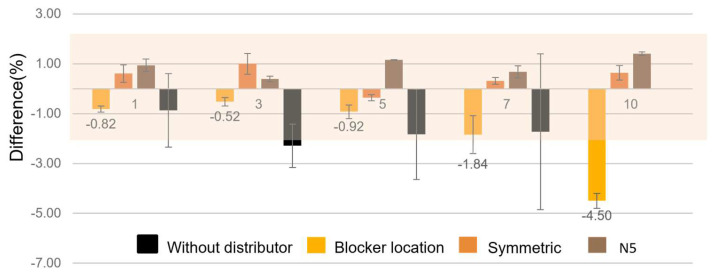
Differential analysis of simulated blood viscosity measurements between the high-flow-rate flow field stabilizer design and the standard viscometer. Data represent mean ± standard deviation; *n* = 5.

The degree of flow field uniformity improvement was quantitatively assessed, as shown in Figure 9c. The incorporation of flow distributors led to a 15.44% enhancement in flow field symmetry compared to the baseline condition. Although the four tested designs exhibited similar levels of improvement, slight variations were still observed. Further analysis of Figure 9a reveals distinct flow deviation patterns at the chip inlet region following the outlet of each flow distributor. These velocity trends are consistent with the predictions from the earlier pre-simulations. Among all tested designs, the N5 configuration exhibited the least flow non-uniformity at the chip inlet. As the fluid progresses along the microchannel, the influence of the flow distributor gradually diminishes, leading to convergence in performance among the different configurations. This trend explains the relatively close compensation values observed in Figure 10. Based on the comparison, the flow field stabilization effectiveness follows the order, N5 > Symmetric ≈ Porosity 0.25 > Blocker position.

Based on these results, the N5, Symmetric, and Blocker position designs are selected for further prototyping. The Porosity-adjusted configuration will not be fabricated due to the potential risk of vortex formation associated with extreme narrowing. The selected designs are expected to enhance stability during viscosity measurement and facilitate future applications involving high-flow-rate operation. For context, a comparison of representative viscosity measurement techniques is summarized in Table 2.

### 3.4. Implementation of Flow Distributors

To evaluate the effectiveness of the flow field stabilizer, simulated blood was used as the test fluid. Except for the addition of the stabilizer structure, the chip design remained identical to the previously optimized Design B, which was tailored for a specific shear rate. In this experiment, the performance of the stabilizer was assessed by comparing the measurement results from the stabilizer-integrated chip with those from the original chip without the stabilizer, using the simulated blood sample. This comparison allows for a direct evaluation of the stabilizer’s influence on flow field stability under practical conditions.

Although microchannel geometric parameters influence the local shear-rate distribution, they do not directly determine the viscosity value extracted by the proposed method. In this study, viscosity is defined as an effective parameter derived from the established relationship between flow rate ratio, velocity field, and interface position under controlled boundary conditions. The microchannel geometry is fixed and calibrated in both simulation and experiments, ensuring that the extracted viscosity reflects the intrinsic fluid property rather than geometric artifacts. The validity of this standard is further confirmed through consistency between numerical predictions and experimental measurements.

As shown in Figure 10, the original chip design without a stabilizer maintained a viscosity measurement accuracy of approximately 98% for simulated blood under standard flow conditions. However, as the flow rate ratio increased, the standard deviation of repeated measurements also rose significantly. Since standard deviation directly reflects the measurement stability across replicates, this trend indicates that the original chip’s performance deteriorates under high-flow conditions. Consequently, the measurable viscosity range of the chip is inherently limited, as the measurable range is directly proportional to the applied flow rate ratio.

With the introduction of flow field stabilizer structures, all three tested designs showed a notable reduction in standard deviation under repeated measurements. Specifically, under high flow rate ratios, the standard deviation was reduced by at least 50% compared to the initial design, confirming the effectiveness of the stabilizers.

Each of the three designs also influenced measurement accuracy differently. The first design, which adjusted the blocker position, exhibited a gradual decline in measured viscosity as the flow rate ratio increased. This trend is consistent with the simulation results discussed in previous section, where the asymmetric velocity field intensified with increasing flow rate ratio, leading to biased flow distribution. In practical testing, the measurement error exceeded 2% when the flow rate ratio surpassed 7, limiting the effective operating range of this design to a narrow region. Minor discrepancies between experimental results and CFD predictions can be attributed to several factors, including flow-rate fluctuation from syringe pumps, fabrication-induced channel asymmetry, and temperature-dependent viscosity variations that are not fully captured in the idealized numerical model. In addition, the CFD simulations assume perfectly developed laminar flow and uniform boundary conditions, whereas experimental conditions inevitably introduce small perturbations. Despite these factors, the overall trends and relative improvements in flow stability show good agreement, supporting the validity of the proposed design.

The symmetric design maintained a measurement error below 2% across all tested flow rate ratios, demonstrating excellent stability and accuracy throughout.

The flow path number adjustment design also maintained measurement error within 2% regardless of the flow rate ratio. Although the measured viscosity values were consistently slightly higher than those obtained using the standard viscometer, the deviations appeared random and exhibited no significant trend, suggesting a systematic yet stable offset.

Overall, the flow stabilizer performance under actual measurement conditions followed the order, N5 ≈ Symmetric > Blocker position, which is different from the simulation-predicted trend of N5 > Symmetric > Blocker position. This minor discrepancy may be attributed to the fact that both the N5 and symmetric designs performed equally well under the flow rate ratio of 10, making them difficult to distinguish experimentally. The blocker position design, on the other hand, consistently ranked lower in both simulation and experimental results. Based on these findings, the N5 and symmetric designs will be selected for subsequent experiments.

### 3.5. Implementation of Milk Formation Time

The accuracy of viscosity measurement in microfluidic systems is strongly affected by temperature and pump precision. Even a 1 °C drift can alter viscosity by several percent, while pulsation from syringe pumps may cause transient shear-rate fluctuations that enlarge SD. To mitigate these effects, temperature stabilization and calibrated constant-flow operation were implemented prior to each run. Such control measures are essential to achieving the low SD values summarized in Table 3.

**Figure 11:**
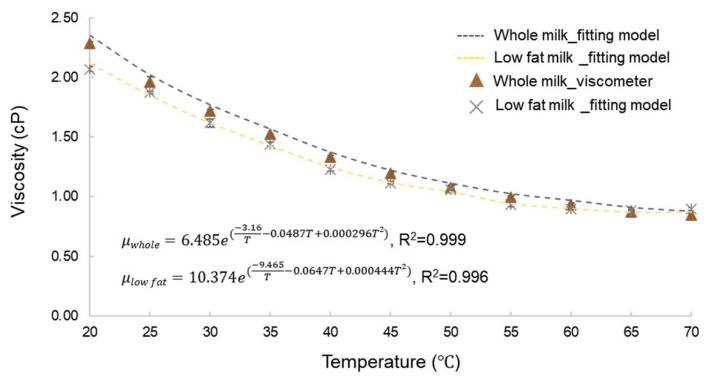
Viscosity–temperature relationship of whole milk and low-fat milk. The data were fitted using a multi-parameter Arrhenius-type equation, with R^2^ values exceeding 0.99 for both models.

To better understand the characteristics of the milk samples, we first examined the temperature-dependent viscosity trend as shown in Figure 11. As the temperature increased gradually from room temperature to conditions representative of low-temperature pasteurization, the viscosity of milk was observed to decrease progressively [43].

The microfluidic chip with an integrated flow field stabilizer exhibited viscosity measurement accuracy comparable to that of a standard viscometer as shown in Table 2 and Table 3. At lower flow rate ratios, however, the N5 design demonstrated slightly better measurement stability, as indicated by a lower standard deviation compared to the Symmetric design. Given that the subsequent milk gelation experiments were conducted under low flow rate ratio conditions, the N5 chip was selected for viscosity monitoring.

**Figure 12:**
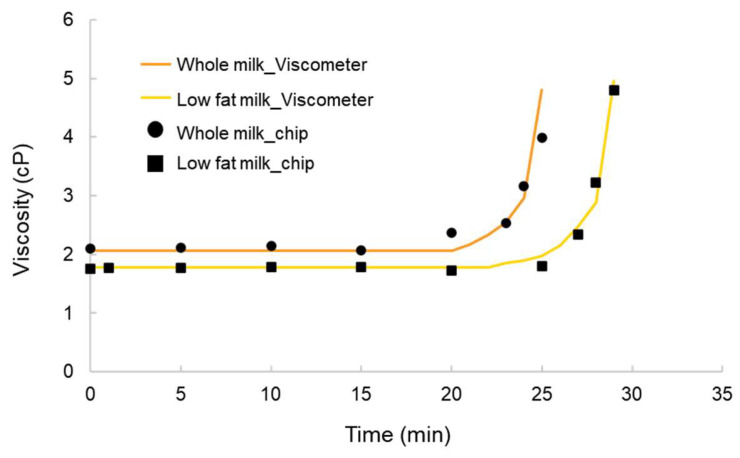
Viscosity–time profiles of whole milk and low-fat milk during gelation, measured using both the microfluidic chip and a standard viscometer.

The experimental results of the milk gelation process are shown in Figure 12. During the tests, the viscosity of whole milk increased from an initial value of approximately 2 cP to 4 cP, representing a twofold increase. This doubling of viscosity was used as an indicator of the onset of gelation and defined as the gelation initiation time. According to the viscometer measurements, the gelation times for whole milk and low-fat milk were 24.5 min and 28.5 min, respectively. When measured using the microfluidic chip developed in this study, the corresponding gelation times were 25 min and 28.5 min, showing excellent agreement with the reference values. This confirms the feasibility and accuracy of the chip platform in monitoring the milk gelation process.

Moreover, the chip-based viscosity sensing approach demonstrated good suitability for food-based samples (e.g., milk) and holds potential for use with other types of fluids, such as biological fluids, pharmaceutical solutions, and industrial liquids. These findings highlight the platform’s broad application potential in fields such as food engineering, biomedical diagnostics, and process monitoring.

## 4. Conclusions

The high-flow-rate flow field stabilizer significantly improved the stability of flow conditions within the microfluidic chip across various sample types. As a result, the viscosity measurement range was successfully expanded from 1–10 cP to 1–50 cP. In comparison with conventional rheometers and sensor-based viscometers summarized in Table 2, the proposed microfluidic platform uniquely combines small sample demand (<1 mL), high precision (>95%), and broad viscosity coverage (1–50 cP) while maintaining low SD (2–7%). In repeated measurements using simulated blood samples, the SD was reduced by over 50% compared to the original design, demonstrating a substantial enhancement in measurement stability and reproducibility [43].

This significant reduction in measurement variability confirms the stabilizer’s effectiveness in achieving stable co-flow conditions even at high flow rate ratios.

Consequently, the platform offers an advantageous balance between accuracy, repeatability, and operational simplicity, positioning it as a practical tool for both biomedical and food-engineering applications.

These findings confirm that the high-flow-rate stabilizer effectively resolves the issue of non-uniform flow distribution under high-flow conditions observed in the initial design, thereby greatly improving measurement reliability. When applied to milk formation experiments, the chip enabled monitoring of viscosity changes during the transition of milk into dairy products. The resulting viscosity profiles closely matched those obtained using a standard viscometer, validating the accuracy and feasibility of the microfluidic viscosity sensing approach. The platform developed in this study combines a high-flow-rate stabilizer, overcoming key limitations of previous design. With only 4% of the sample volume required, the chip achieves viscosity measurement error below 5% and overall accuracy exceeding 95%, enabling high-precision, low-volume, and viscosity monitoring of non-Newtonian fluids.

The proposed system demonstrated the performance and applicability in both simulated blood viscosity monitoring and dairy production processes, offering a promising new solution for fluid property analysis. The results presented here may support future developments in applying microfluidic technology to clinical, food, and industrial fields, particularly in enhancing measurement precision and enabling integration into smart sensing systems

## Figures and Tables

**Table 1:** COMSOL simulation parameters and settings.

Setting	Value	Unit
Inelastic model	Power law	module
Multi-Physical model	Transport of Diluted Species	module
Sample fluid	Simulating blood	cP
Reference fluid	DI water	cP
Flow rate 1 (Sample input)	10	μl/min
Flow rate 2 (Reference input)	10	μl/min
Diffusion coefficient	$10^{-11}$	m^2^/s

**Table 2:** Comparative performance of representative viscosity measurement techniques, including accuracy, measurable range, sample volume, and standard deviation (SD).

Measurement Method	Advantage	Sample Limitation	Accuracy (%)	SD	Functional Range (cP)	Resolution (cP)	Shear Rate (s^−1^)	Ref
Vibration viscometer	Simple operation	Newtonian fluid, ≥20 mL sample	95%	<0.2%	0.4–1000	0.2	-	[37]
Magnetoelastic Sensor	Quick, low-cost measurement	Newtonian fluid, ≥100 μL sample	95%	*n* = 5, <1%	1–10	0.001	-	[38]
Rheometer	High precision, High accuracy	Non-Newtonian fluid, ≥25 mL sample	95–98%	*n* = 5, <3%	1–10^7^	0.1	$\geq 10^{-3}$	[39]
Pressure sensor	The sensitivity was better than 0.5 cP	Non-Newtonian fluid	81.50%	*n* = 5, <2%	2–100	0.5	20–345.1	[40]
Two-phase microfluidic viscometer	Simultaneously measured the multiple samples	Non-Newtonian fluid, prepare: 1.5 mL	92%	*n* = 6, 1–3%	1–10,000	1	1-6000	[41]
	Less than 10 μL sample requirement	Non-Newtonian fluid, ≥10 μL	95%	<2%	1–10	0.1	20,000–40,000	[42]
	Low shear rate, High accuracy	Non-Newtonian fluid, prepare: ≥1 mL, waste: ≥120 uL	95%	*n* = 3, 2–7%	1–10	0.01	10–100	[17]
	Low shear rate, High accuracy	Non-Newtonian fluid, prepare: ≥1 mL, waste: ≥120 uL	95%	*n* = 3, <1%	1–10	0.01	10–100	This study

**Table 3:** Viscosity of whole and low-fat milk measured using the Symmetric and N5 chip designs under different flow rate ratios. Values are mean ± SD (cP, n = 5).

Milk Type	Chip Design	FR = 1	3	5	7	10
Whole milk	Symmetric	1.996 ± 0.066	2.021 ± 0.023	1.997 ± 0.003	1.995 ± 0.003	1.970 ± 0.003
	N5	2.005 ± 0.021	1.997 ± 0.003	2.005 ± 0.008	2.000 ± 0.014	2.013 ± 0.076
Low-fat milk	Symmetric	1.682 ± 0.011	1.701 ± 0.002	1.704 ± 0.004	1.712 ± 0.006	1.743 ± 0.003
	N5	1.709 ± 0.006	1.715 ± 0.010	1.722 ± 0.024	1.683 ± 0.006	1.759 ± 0.001

## Data Availability

The authors declare no data availability.
